# A prospective study to compare serial changes in pain scores for patients with and without a history of frequent ED utilization

**DOI:** 10.1016/j.heliyon.2021.e07216

**Published:** 2021-06-06

**Authors:** Ryan Joseph, Alainya Tomanec, Thomas McLaughlin, Jose Guardiola, Peter Richman

**Affiliations:** a Department of Emergency Medicine, University of Texas Health Science Center at San Antonio, San Antonio, TX, USA; b Department of Emergency Medicine, CHRISTUS Health/Texas A&M, Corpus Christi, TX, USA; c Department of Mathematics, Texas A&M-Corpus Christi, Corpus Christi, TX, USA

**Keywords:** Pain, Analgesics, Visual analog scale, Pain scores, Pain medications, Emergency department, Opioids, Opioid epidemic, Patient satisfaction

## Abstract

**Background:**

In the face of the opiate addiction epidemic, there is a paucity of research that evaluates limitations for our current pain rating methodologies for patient populations at risk for drug seeking behavior.

**Objective:**

We hypothesized that VAS scores would be higher and show less serial improvement for patients with a history of frequent ED use.

**Methods:**

This was a prospective, observational cohort study of a convenience sample of adult ED patients with chief complaint of pain. Initial VAS scores were recorded. Pain scores were subsequently updated 30–45 min after pain medication administration. ED frequenter defined as having >4 ED visits over a 1-year time period. Categorical data analyzed by chi-square; continuous data analyzed by t-tests. A multiple linear regression performed to control for confounding.

**Results:**

125 patients were enrolled; 51% ED frequenters. ED frequenters were similar to non-ED frequenters with respect to gender, mean age, Hispanic race, educational level, chief complaint type, and initial pain medication narcotic. ED frequenters more likely to have higher initial VAS score (9.17+/-1.25 vs. 8.51+/-1.68; p = 0.01) and higher second VAS scores (7.48+/-2.56 vs. 5.00+/-3.28; p <0.001) and significantly lower mean change in first to second VAS scores (1.69+/-2.17 vs. 3.51+/-3.25; p <0.001). Within our multiple linear regression model, only ED frequenter group (p < 0.001) and private insurance status (0.04) were associated with differences in mean reduction in pain scores.

**Conclusion:**

We found that ED frequenters had significantly less improvement between first and second VAS measurements.

## 1 Introduction

Pain is one of the most commonly encountered chief complaints in the emergency department [1]. In 2015, the Center for Disease Control identified abdominal pain as the leading cause of emergency department (ED) presentation (8.8%), followed by chest pain (5.3%) and headache (2.8%), with painful conditions in aggregate comprising nearly 20 percent of all ED visits [2].

With this backdrop, regulatory agencies including the Joint Commission on the Accreditation of Healthcare Organizations (JCAHO) and Centers for Medicare and Medicaid (CMS) have increasingly emphasized pain management in their assessment of health facility and provider competence. In 2012, CMS created an “Incentive Fund” which partially based hospital reimbursement off of the Hospital Consumer Assessment of Healthcare Providers and Systems (HCAHPS) patient satisfaction survey which directly asks patients how well their pain was controlled [3].

While attempting to improve the comfort of patients in pain, it is less clear what role regulatory forces may have unwittingly served in leading physicians to overprescribe narcotics. Unintentional opioid overdoses have now become a national epidemic with the majority being related to prescription oral forms, surpassing motor vehicle crashes as the leading cause of death in the United States [4]. In 2008, drug overdoses were responsible for 20,044 deaths, and 73.8% of those involved opioids [4].

Competing pressures to control pain in the face of a growing opioid addiction epidemic place emergency physicians in a precarious position for many of the patients treated in the ED. Oligoanalgesia may lead to diminished reimbursement and lower satisfaction scores with potential administrative ramifications, while overly generous prescribing may have regulatory consequences for the physician [5, 6]. Emergency physicians are hampered further in balancing this dilemma by the fact that pain/pain reporting is a subjective experience, and we are currently lacking well-developed tools to identify patients with reported acute pain who are abusing the ED for secondary gain/exhibiting drug seeking behavior. Current approaches to evaluate physicians and institutions for satisfactory pain management do not take such factors into account.

Relevant to this concern, investigators have previously described that there are significant pain score reporting differences between subsets of ED patients [7, 8]. For example, Raftery et al. described gender differences for reporting of pain and physician perception of pain [8]. Likewise, utilizing a post-care survey instrument, Todd et al. found that there were disparities for reported pain levels between patients with chronic pain patients as compared with those experiencing acute recurrent pain [9]. Although the survey investigated the overall adequacy of pain relief reported by the patients, the investigators did not attempt to specifically measure and assess initial vs. subsequent scoring of pain in the ED.

As a consequence of poor access to alternative sites for pain and other medical care, emergency physicians encounter a large number of patients who utilize the emergency department frequently. Our literature search did not reveal prior studies that evaluate whether or not frequent users of the ED are a higher risk to have poor response to analgesic therapy. In view of this knowledge gap, we initiated a prospective study to examine the relationship between frequent ED utilization and patients' reports changes in pain score during serial assessments.

We hypothesized that VAS scores would be higher and show less serial improvement as reported by patients with a history of frequent ED use.

## 2 Methods

### 2.1 Study design

We conducted a prospective, observational cohort study during a nine-month period from January 5, 2017 through September 28, 2017.

### 2.2 Setting

The study was based in the ED of a 275-bed community hospital with an annual census of approximately 45,000 patient visits. The study was approved by our hospital's Institutional Review Board prior to the initiation of data collection.

### 2.3 Study population

Patients presenting with a chief complaint of abdominal pain, back pain, flank pain, or headache were consented for enrollment at point of care by emergency physicians with the aid of trained research associates (convenience sample). We excluded pregnant females, prisoners, patients less than 18 years old, or any patient that ultimately was admitted and required a surgical intervention for their condition. Consistent with the methods of Locker et al., we defined an ED frequenter as a patient who within a 1-year period look-back from the current visit was found to have greater than or equal to 4 visits to any one of the 6 EDs within our southern Texas hospital system [10]. Prior ED utilization was determined by structured review of our electronic medical record that catalogs all system encounters by type of setting.

### 2.4 Study protocol

Consenting participants completed a brief survey providing demographic, chief complaint and historical information. We recorded pretreatment pain scores that were reported by patients to their treating physician based on a 0–10 visual analog scale (VAS). Pain medication was administered at the discretion of the treating physician, and, within 30–45 min of medication administration another pain score reported to the treating physician was recorded on the same scale previously utilized. If subsequent VAS scores were taken during the patients stay in the ED, these were also recorded.

### 2.5 Statistical analysis

The Anderson-Darling test was utilized to assess the data for normality. Continuous data are presented as frequency of occurrence and were analyzed by t-tests (parametric data) or the Wilcoxon rank sum test (non-parametric data). Categorical data are presented as a means +/- standard deviation and were analyzed using chi-square. Alpha was set at 0.05. JMP v14 SAS Institute software was utilized for the analysis. We performed a multiple linear regression to control for possible confounding. The dependent variable was the change in initial VAS scores, and we included in the multiple regression model 7 main factors (patient characteristics/demographics in Table 1) and two cross effects. The method used was backwards elimination and the criteria was minimizing BIC.

**Table 1:** Study group characteristics.

Male	40% (75)
Hispanic	81% (101)
Household Income <20K	73% (90)
Less than High School Education	33% (41)
Private Insurance	25% (31)
ED Frequenter	51% (64)

The primary outcome parameter was to compare the serial improvement in pain scores between ED non-frequenters and frequenters. For the study to have 80% power with alpha set at 0.05, we calculated an a priori sample size of 46 in each comparison group assuming a 2.5 mean change in pain score for non-frequenters vs. 1.5 mean change for frequenters.

## 3 Results

125 patients were enrolled from January 5, 2017 through September 28, 2017. We identified 51% of our patients as ED frequenters. The most common chief complaint was abdominal pain, which accounted for 81% of ED visits in our study. Table 1 summarizes the characteristics of the study group that was predominantly Hispanic and from lower socioeconomic and educational status.

Table 2 summarizes characteristics of ED frequenters vs. non-ED Frequenters, and, for most variables the two groups were similar. ED frequenters were more likely to have an income of <$20,000 when compared to non-ED frequenters (84% vs 62%, p = 0.01) and less likely to have private insurance when compared to ED non-frequenters (14% vs 36%, p = 0.01). The two groups were similar with respect to the distribution of chief complaint types (p = 0.68), and there was no statistical difference in the frequency for which they received an initial dose of narcotics (71% vs. 79%; p = 0.26).

**Table 2:** Comparison of ED Frequenter vs. non-ED Frequenter Patients.

	ED Frequenters	Non ED Frequenters	P value	95% CI difference for Frequenters vs non Frequenters
Male	42%	38%	0.61	Males Fr – Males NFr (-13%, 22%)
Female	58%	62%	0.61	
Hispanic	77%	85%	0.22	Hisp Fr – His NFr (-22%, 5%)
Non-Hispanic	23%	15%	0.22	
Household Income <20K	84%	62%	0.01	HI <20k Fr – HI <20k Nfr (6%,37%)
Household Income >20K	16%	38%	0.01	
Private Insurance	15%	36%	0.01	PI Fr – NPI NFr (-36%, -7%)
No Private Insurance	85%	64%	0 .01	
Less than High School Education	31%	36%	0.52	LHs Fr – LHs NFr (-22%, 11%)
At least High School Education	69%	64%	0.52	

ED frequenters were more likely to have higher initial VAS scores (9.17 +/- 1.25 vs 8.51 +/- 1.68; p = 0.01), higher second VAS scores (7.48 +/- 2.56 vs 5.00 +/- 3.28; p < 0.001), and lower mean change from 1st to 2nd VAS scores (1.69 +/- 2.17 vs 3.51 +/- 3.25; p < 0.001) compared to patients who did not frequently visit the ED. 32 patients (22 frequenters) received a second dose of analgesic. There were no significant differences in mean pain score change for ED frequenters vs. non-frequenters (2.6+/-3.1 vs 1.8+/-3.1; p = 0.28).

To control for possible confounding, we performed a multiple linear regression that included all clinical and historical variables collected for the study. Within our model, the association between ED frequenter status and mean pain score reduction remained significant (p < 0. 001). With respect to other patient characteristics, only private insurance status was associated with differences in mean reduction in pain scores (p = 0.04).

## 4 Discussion

In this prospective, observational cohort study, we sought to determine if patients that frequently utilize the ED (ED frequenters) had less serial change in their VAS scores after pain management when compared to patients without a pattern of high ED utilization. We found that ED frequenters were significantly more likely to have higher initial VAS scores, higher second VAS scores, and less serial improvement in their VAS scores following analgesic administration.

Our findings raise some new concerns for regulators and administrators who wish to assess the adequacy by which emergency physicians treat their patients reporting pain. If our results are validated in other populations, we have identified a subset of patients for which there is lower likelihood that the provision of analgesics will result in end-of-visit improvements. Unfortunately, current evaluation methods do not control for such variables both within institutions and between institutions respectively. Thus, EDs with higher recidivism rates may be at risk for scoring poorly on key pain control and patient satisfaction metrics based on census characteristics that are, clearly, outside the sphere of physician influence.

Investigators previously have evaluated the assessment and treatment of pain in the ED from a variety of perspectives 1, 5, 6, 11, 12, 13, 14, 15, 16, 17, 18, 19, 20, 21, 22, 23, 24, 25, 26, 27, 28, 29, 30, 31, 32, 33, 34, 35, 36, 37, 38, 39, 40, 41, 42, 43, 44, 45, 46, 47, 48, 49, 50, 51, 52, 53, 54, 55, 56 Several have evaluated serial assessments of pain and noted a frequent lack of improvement and/or satisfaction despite treatment [11, 12, 13] Todd et al. conducted a telephone survey of 500 adult patients with either chronic or recurrent pain who reported an ED visit within the past two years [11]. Less than half the patients in both groups reported following treatment that they felt “complete” or “a great deal” of pain relief. While analogous to our study for evaluating patient subgroups that would appear to be at risk for poor treatment response, the authors did not compare those patients to a subgroup that would appear to be at less risk for poor treatment response such as the non-frequenter group we considered. Further, the retrospective method of the survey, well after the point of actual care, provides risk of recall bias by the patient that our prospective, serial reassessment/recording of pain scores avoids.

Noting the poor treatment improvement in our ED frequenter study group and that observed by Todd et al. for chronic and recurring pain sufferers, we can speculate that these and other patient subgroups likely contribute significantly to the observations by other investigators that interventions to improve pain management often do not result in increasingly satisfied patients [12, 13]. In the context of acute musculoskeletal injury, Sturesson et al. surveyed 80 patients before and 80 patients after the implementation of mandatory pain documentation in their ED [12]. They found that, while there was a significant difference in the number of patients receiving analgesics (41% pre-intervention vs 68% post-intervention; (p < 0.003)) as well as reduced reported pain intensity at discharge (p < 0.03), patients did not provide improved satisfaction scores post-intervention.

As much as patient characteristics may play a role in ED pain management challenges, Sampson et al. explored ED staff factors that might contribute to poor satisfaction reporting by patients despite recorded serial pain score improvements [13]. They collected data from 3 case study EDs including 143 h of non-participant observation, 37 ED staff interviews, and 19 patient interviews. They observed differences between EDs and staff as to whether the pain score was documented directly by the patient or formulated by the clinician. Often the staff were reluctant to accept patient-reported scores if inconsistent with their own assessment of the patient pain level, and this was particularly exacerbated when the score was used as a tool for auditing appropriate pain management.

Our findings and the aforementioned reports outline significant challenges for emergency physicians when one considers the significant potential for patient and staff factors outside of the physician domain of control to influence pain score reporting and satisfaction. We believe additional research is warranted to validate our findings and to identify additional patient characteristics that may be associated with low likelihood of serial pain score reporting improvement.

## 5 Limitations

Our study has several limitations that warrant discussion. First of all, we did not enroll patients consecutively, and we did not track patient refusals to participate, which raises concern for selection bias. We attempted to mitigate this risk by utilizing research associates who had highly varied service hours that crossed all days and times of the week. ED frequenter and non-frequenter groups within our study sample were found to be similar across most characteristics. We acknowledge that our patient population, which has a high proportion of Hispanics from lower socioeconomic status, is dissimilar from that observed in other settings/regions of the country, thus, raising external validity concerns. However, in our regression model to attempt to control for confounding, we did not find any association between our primary outcome parameter and the variables race, income, and educational level.

Another limitation of our investigation was the lack of a standardized treatment protocol for patients in the two comparison groups. Physicians, at their discretion, treated patients with analgesics and chose the type of medication provided. Fortunately, we found that patients in the two groups were provided narcotics with similar frequency suggesting some degree of uniformity in therapy. Physicians and patients alike were not blinded to the medication administered, but it is unclear how blinding might have impacted our outcome parameter when such a high percentage of patients in both groups received narcotics.

We also allowed for a broad array of patients with a variety of chief complaints to be enrolled. However, we note that the distribution of chief complaints was not significantly different between ED frequenters and non-frequenters. Thus, we do not believe that inclusion of a diverse group of patients with pain confounded our results. We do think future investigators should consider larger studies that focus on patients solely with a specific type/location of pain (e.g. musculoskeletal back pain) to evaluate whether our findings are consistent across chief complaints.

Finally, while our results seem to support a widely held belief by emergency physicians that patients who frequently visit the emergency department are more likely to not improve with initial analgesic administration, our study does not specifically identify factors that contribute to this phenomenon. Grover et al. observed, “drug seeking patients exhibit classically described drug-seeking behavior (e.g. requesting refill of an analgesic prescription) with only low to moderate frequency [57]. Thus, it may be difficult for future investigators to attribute frequent visits necessarily with patients who are drug seeking.

## 6 Conclusion

We found that ED frequenters had significantly less improvement between first and second VAS measurements than those observed for patients who had prior history of infrequent visits. Future investigators should conduct studies to confirm our findings in other settings as well as to identify other high-risk groups/patient characteristics for poor reported pain treatment response.

## Declarations

### Author contribution statement

Ryan Joseph and Peter Richman: Conceived and designed the experiments; Performed the experiments; Analyzed and interpreted the data; Wrote the paper.

Alainya Tomanec and Thomas McLaughlin:Conceived and designed the experiments; Performed the experiments; Wrote the paper.

Jose Guardiola: Conceived and designed the experiments; Analyzed and interpreted the data; Wrote the paper.

### Funding statement

This research did not receive any specific grant from funding agencies in the public, commercial, or not-for-profit sectors.

### Data availability statement

Data will be made available on request.

### Declaration of interests statement

The authors declare no conflict of interest.

### Additional information

No additional information is available for this paper.

## References

[1] Cordell W.H., Keene K.K., Giles B.K., Jones J.B., Jones J.H., Brizendine E.J. (2002). The high prevalence of pain in emergency medical care. Am. J. Emerg. Med..

[2] U.S Department of Health and Human Services. Centers for Disease Control and Prevention National hospital ambulatory medical care survey: 2015 emergency department summary tables. https://www.cdc.gov/nchs/data/nhamcs/web_tables/2015_ed_web_tables.pdf.

[3] Zusmann E.E. (2012). HCAHPS replaces press ganey survey as quality measure for patient hospital experience. Neurosurgery.

[4] Paulozzi L.J. (2006). Opioid analgesic involvement in drug abuse deaths in American metropolitan areas. Am. J. Publ. Health.

[5] Bhakta H.C., Marco C.A. (2014). Pain management: association with patient satisfaction among emergency department patients. J. Emerg. Med..

[6] Marco C.A., Davis A., Chang S., Mann D., Olso J.E. (2015 Nov). ED patient satisfaction: factors associated with satisfaction with care. Am. J. Emerg. Med..

[7] Todd K.H., Ducharme J., Choiniere M. (2007). Pain in the emergency department: results of the pain and emergency medicine initiative (PEMI) multicenter study. J. Pain.

[8] Raftery K.A., Smith-Coggins R., Chen A. (1995). Gender associated difference in emergency department pain management. Ann. Emerg. Med..

[9] Todd Knox H. (2005). Chronic pain and aberrant drug related behavior in the emergency department. J. Law Med. Ethics.

[10] Locker T.E., Baston S., Mason S.M. (2007). Defining frequent use of an urban emergency department. Emerg. Med. J..

[11] Todd K.H., Cowan P., Kelly N. (2010 Dec). Chronic or recurrent pain in the emergency department: national telephone survey of patient experience. West. J. Emerg. Med..

[12] Sturesson L., Falk A.C., Castren M. (2016 Oct). Mandatory documentation of pain in the emergency department increases analgesic administration but does not improve patients' satisfaction of pain management. Scand J Pain.

[13] Sampson F.C., O’Cathain A., Goodacre S. (2017. Oct). Whose pain is it anyway? Qualitative research exploring how the 0-10 pain score is used in practice within the adult emergency department. Ann. Emerg. Med..

[14] Rupp T., Delaney K.A. (2004). Inadequate analgesia in emergency medicine. Ann. Emerg. Med..

[15] Fosnocht D.E., Swanson E.R., Barton E.D. (2005). Changing attitudes about pain and pain control in emergency medicine. Emerg. Med. Clin. North Am.

[16] Wilson J.E., Pendleton J.M. (1989). Oligoanalgesia in the emergency department. Am. J. Emerg. Med..

[17] Baumann B.M., Holmes J.H., Chansky M.E. (2007). Pain assessments and the provision of analgesia: the effects of a templated chart. Acad. Emerg. Med..

[18] Blankenship J.F., LeGrand Rogers M.D., White J. (2012). Prospective evaluation of the treatment of pain in the ED using computerized physician order entry. Am. J. Emerg. Med..

[19] Boyd R.J., Stuart P. (2005). The efficacy of structured assessment and analgesia provision in the paediatric emergency department. Eng. Manag. J..

[20] Campbell P., Dennie M., Dougherty K. (2004). Implementation of an ED protocol for painmanagement at triage at a busy level I trauma center. J. Emerg. Nurs..

[21] Clere F., Leclerc G., Soriot S. (2001). Intravenous drugs for the management of acute pain: contribution of a written protocol for emergency care units. [French]. Douleurs.

[22] Corwin D.J., Kessler D.O., Auerbach M. (2012). An intervention to improve pain management in the pediatric emergency department. Pediatr. Emerg. Care.

[23] Crocker P.J., Higgingbottom E., King B.T. (2012). Comprehensive pain management protocol reduces children's memory of pain at discharge from the pediatric ED. Am. J. Emerg. Med..

[24] Day F., Hoang L.P., Ouk S. (1995). The impact of a guideline-driven computer charting system on the emergency care of patients with acute low back pain. Proceedings - the Annual Symposium on Computer Applications in Medical Care.

[25] Decosterd I., Hugli O., Tamches E. (2007). Oligoanalgesia in the emergency department: short-term beneficial effects of an education program on acute pain. Ann. Emerg. Med..

[26] Doherty S., Knott J., Bennets S. (2012). National project seeking to improve pain management in the emergency department setting: findings from the NHMRC-NICS National Pain Management Initiative. Emerg. Med. Australasia.

[27] Eisen S.Amiel K. (2007). Introduction of a paediatric pain management protocol improves assessment and management of pain in children in the emergency department. Arch. Dis. Child..

[28] Ender K., Freed J., Babineau J. (2010). Improvement in acute management of vaso-occlusive pain in pediatric sickle cell Disease with use of a clinical pathway. Blood.

[29] Fosnocht D.E., Swanson E.R. (2007). Use of a triage pain protocol in the ED. Am. J. Emerg. Med..

[30] Gawthorne J., Welchi S., Robertson F. (2010). Implementation of a guideline to improve prescription of analgesia for adult trauma patients in an Emergency Department. Aust. Emerg. Nurs. J..

[31] Goodacre S.W., Roden R.K. (1996). A protocol to improve analgesia use in the accident and emergency department. J. Accid. Emerg. Med..

[32] Hawkes C., Kelleher G., Hourihane J. (2008). Paediatric analgesia in an emergency department. Ir. Med. J..

[33] Iyer S.B., Schubert C.J., Schoettker P.J. (2011). Use of quality-improvement methods to improve timeliness of analgesic delivery. Pediatrics.

[34] Jackson S.E. (2010). The efficacy of an educational intervention on documentation of pain management for the elderly patient with a hip fracture in the emergency department. J. Emerg. Nurs..

[35] Jadav M.A.R., Lloyd G., McLauchlan C. (2009). Routine pain scoring does not improve analgesiaprovision for children in the emergency department. Eng. Manag. J..

[36] Jones J.B. (1999). Assessment of pain management skills in emergency medicine residents: the role of a pain education program. J. Emerg. Med..

[37] Kaplan C.P., Sison C., Platt S.L. (2008). Does a pain scale improve pain assessment in the pediatric emergency department?. Pediatr. Emerg. Care.

[38] Kelly A.M. (2000). A process approach to improving pain management in the emergency department: development and evaluation. J. Accid. Emerg. Med..

[39] Kelly A.M. (2000). Nurse-managed analgesia for renal colic pain in the emergency department. Aust. Health Rev..

[40] Kuan S.C., Collins N.C., Ryan J.M. (2010). Treating pain in the emergency department. Eur. J. Emerg. Med..

[41] Le May S., Johnston C., Choiniere M. (2009). Pain management practices in a pediatric emergency room (PAMPER) study: interventions with nurses. Pediatr. Emerg. Care.

[42] Muntlin A., Carlsson M., Safwenberg U. (2001). Outcomes of a nurse-initiated intravenous analgesic protocol for abdominal pain in an emergency department: a quasi-experimental study. Int. J. Nurs. Stud..

[43] Nelson B.P., Cohen D., Lander O. (2004). Mandated pain scales improve frequency of ED analgesic administration. Am. J. Emerg. Med..

[44] Rogovik A.L., Rostami M., Hussain S. (2007). Physician pain reminder as an intervention to enhance analgesia for extremity and clavicle injuries in pediatric emergency. J. Pain.

[45] Santervas Y.F., Cotanda C.P., Carrelero L.M. (2010). Impact of a program to improve pain management in an emergency department. Eur. J. Emerg. Med..

[46] Somers L.J., Beckett M.W., Sedgwick P.M., Hulbert D.C. (2001). Improving the delivery of analgesia to children in pain. Emerg. Med. J..

[47] Stalnikowicz R., Mahamid R., Kaspi S. (2005). Undertreatment of acute pain in the emergency department: a challenge. Int. J. Qual. Health Care.

[48] Steinberg P., Nangia A.K., Curtis K. (2011). A standardized pain management protocol improves timeliness of analgesia among emergency department patients with renal colic. Qual. Manag. Health Care.

[49] Sucov A., Nathanson A., McCormick J. (2005). Peer review and feedback can modify pain treatment patterns for emergency department patients with fractures. Am. J. Med. Qual..

[50] Tanabe P., Hafner J.W., Martinovich Z. (2012). Adult emergency department patients with sickle cell pain crisis: results from a quality improvement learning collaborative model to improve analgesic management. Acad. Emerg. Med..

[51] Thomas S.H., Andruszkiewicz L.M. (2004). Ongoing visual analog score display improves Emergency Department pain care. J. Emerg. Med..

[52] Vazirani J., Knott J.C. (2012). Mandatory pain scoring at triage reduces time to analgesia. Ann. Emerg. Med..

[53] Williams S., Holzhauser K., Bonney D. (2012). Improving pain management of abdominal pain in children presenting to the paediatric emergency department: a pre-post interventional study. Aust. Emerg. Nurs. J..

[54] Wong E.M.L., Chan H.M.S., Rainer T.H. (2007). The effect of a triage pain management protocol for minor musculoskeletal injury patients in a Hong Kong emergency department. Aust. Emerg. Nurs. J..

[55] Yanuka M., Soffer D., Halpern P. (2008). An interventional study to improve the quality of analgesia in the emergency department. CJEM.

[56] Guru V., Dubinsky I. (2000). The patient vs. caregiver perception of acute pain in the emergency department. J. Emerg. Med..

[57] Grover C.A., Elder J.W., Close R.J., Curry S.M. (2012 Nov). How frequently are “classic” drug-seeking behaviors used by drug-seeking patients in the emergency department?. West. J. Emerg. Med..

